# Supplementary material for: Characterization of the cell division-associated peptidoglycan amidase AmiA of Chlamydia trachomatis
Source: J Bacteriol. 2026 Mar 11;208(4):e00453-25. doi: 10.1128/jb.00453-25 (PMC13104611; doi:10.1128/jb.00453-25)
Supplement: Supplemental figures — Figures S1 and S2. [file jb.00453-25-s0001.docx]

**Supplemental Figures**

**Suppl. figure 1: Amino acid sequence alignment of AmiA homologs of *C. trachomatis, C. pneumoniae* and *E. coli*.** Red: active site residues. Orange: Identical residues. Blue: PBP motifs. Green: blocking helix. Yellow: interaction helix. Below the alignment, asterisks (*) indicate fully conserved residues between input sequences. Colons (:) indicate conservation of strongly similar properties, while periods (.) indicate conservation of weakly similar properties based on Gonnet PAM 250 matrix used in Clustal Omega alignments.


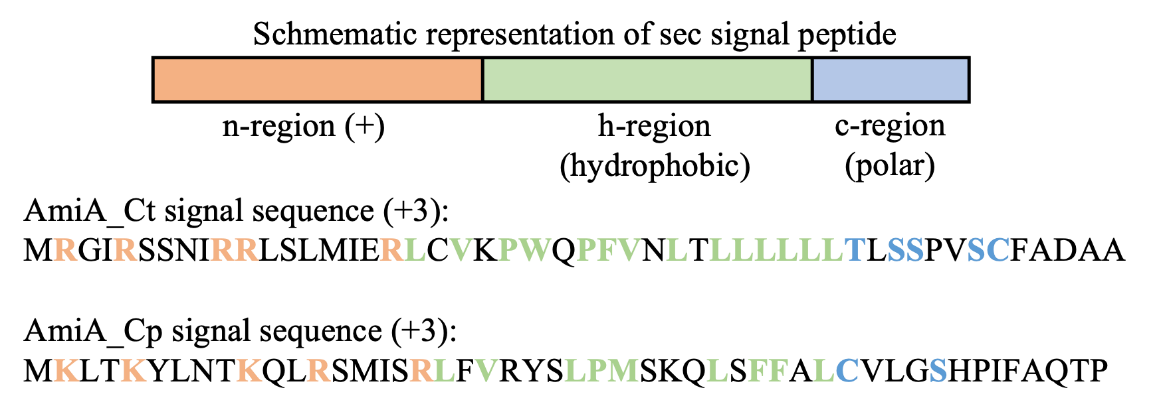


**Suppl. figure 2: Schematic representation of sec-system signal peptides and signal peptide sequences of AmiA_Ct and AmiA_Cp.** Positively charged (+) amino acids in the n-region are marked in red, while hydrophobic amino acids of the h-region are shown in yellow. Polar amino acids of the c-region are depicted in blue. The consensus motif for recognition of the cleavage site by signal recognition particle (SRP) and signal peptidase is A-x-A. Matching amino acids are shown in grey. This suggests a cleavage between residues 47 and 48 in AmiA_Ct, which was also predicted by SignalP 5.0.
