# Supplementary material for: Characterization of the cell division-associated peptidoglycan amidase AmiA of Chlamydia trachomatis
Source: J Bacteriol. 2026 Mar 11;208(4):e00453-25. doi: 10.1128/jb.00453-25 (PMC13104611; doi:10.1128/jb.00453-25)
Supplement: Supplemental tables — Tables S1 to S4. [file jb.00453-25-s0002.docx]

**Supplemental Table 1: Bacterial strains**

| Strain | Genotype | Reference |
| --- | --- | --- |
| *E. coli* ADE24 | Δ *amiA*, *amiB*, *amiC* amidase triple mutant, + pBAD33-*amiC*Ec | [31] |
| *E. coli* C43 (DE3) | F^–^ ompT gal dcm hsdS_B_(r_B_^-^ m_B_^-^)(DE3) | [60] |
| *E. coli* JM83 | rpsL ara Δ(lac-proAB) Φ80dlacZΔM15 | DSM3947 |
| *E. coli* W3110 | F^-^ λ^-^ rph-1 INV(rrnD, rrnE) | DSM5911 |

**Supplemental Table 2: Plasmids**

| Plasmid | Characteristic | Reference |
| --- | --- | --- |
| pASK-IBA-3 | Expression plasmid, AHT-inducible, C-terminal Strep-tag II, Amp^R^ | IBA Lifesciences (Göttingen, Germany) |
| pASK-IBA-3_*ct268* | Expression plasmid, AHT-inducible, C-terminal Strep-tag II, Amp^R^, encodes AmiA_Ct | This work |
| pASK-IBA-3C | Expression plasmid, AHT-inducible, C-terminal Strep-tag II, Cam^R^ | IBA Lifesciences |
| pASK-3C_*ct268* | Expression plasmid, AHT-inducible, C-terminal Strep-tag II, Cam^R^, encodes AmiA_Ct | This work |
| pASK-3C_*ct268*ΔSP | Expression plasmid, AHT-inducible, C-terminal Strep-tag II, Cam^R^, encodes AmiA_CtΔSP | This work |
| pASK-3C_*ct268*_H67A | Expression plasmid, AHT-inducible, C-terminal Strep-tag II, Cam^R^, encodes AmiA_Ct_H67A | This work |
| pASK-3C_*ct268*_H136A | Expression plasmid, AHT-inducible, C-terminal Strep-tag II, Cam^R^, encodes AmiA_Ct_H136A | This work |
| pASK-3C_*ct268*_E205A | Expression plasmid, AHT-inducible, C-terminal Strep-tag II, Cam^R^, encodes AmiA_Ct_E205A | This work |
| pBOMBL-Sa_dCas9vaa:L2 | dCas9-based conditional knockdown plasmid, AHT-inducible, Amp^R^ | [57] |
| pBOMBLCRia(*amiA*) | aTc-inducible CRISPRi-mediated knockdown of *amiA* in *C. trachomatis* L2 | This work |
| pBOMBLCRia-*amiA*_6xH(*amiA*) | aTc-inducible CRISPRi-mediated knockdown of *amiA* in *C. trachomatis* L2, complementation with 6xH-tagged AmiA | This work |
| pBOMBLCRia(NT) | aTc-inducible CRISPRi-mediated knockdown targeted against a random sequence used as a negative control | Hatch and Ouellette, 2023 |
| pET-52b | Expression plasmid, T7 promotor, autoinducible in (DE3)-strains, N-terminal Strep-tag II, Amp^R^ | Novagen **®** (Merck, Darmstadt, Germany) |
| pET-52b_*ct268* | Expression plasmid, T7 promotor, autoinducible in (DE3)-strains, N-terminal Strep-tag II, Amp^R^, encodes AmiA_Ct | This work |
| pET-52b_*ct268*ΔSP | Expression plasmid, T7 promotor, autoinducible in (DE3)-strains, N-terminal Strep-tag II, Amp^R^, encodes AmiA_CtΔSP | This work |
| pET-52b_*ct268*_H67A | Expression plasmid, T7 promotor, autoinducible in (DE3)-strains, N-terminal Strep-tag II, Amp^R^, encodes AmiA_Ct_H67A | This work |
| pET-52b_*ct268_*H136A | Expression plasmid, T7 promotor, autoinducible in (DE3)-strains, N-terminal Strep-tag II, Amp^R^, encodes AmiA_Ct_H136A | This work |
| pET-52b_*ct268_*E205A | Expression plasmid, T7 promotor, autoinducible in (DE3)-strains, N-terminal Strep-tag II, Amp^R^, encodes AmiA_Ct_E205A | This work |

**Supplemental Table 3: Primer sequences**

| Primer | Sequence (5’-3’) | Usage |
| --- | --- | --- |
| *amiA*/(dCas9)/5’ | aagggcgtagcagcataagtaccggaggagaatctATGAGGGGTATCAGATCTTC | Insert *amiA*_6xH into SalI-digested pBOMBLCRia (*amiA*), lower case for plasmid overlap construction |
| *amiA*_6xH/(pLCRia)/3’ | catgagcggatacatatttgaatggttaatggtgatggtgatggtgTTTATGCACTTTTTTTGCTC | Insert *amiA*_6xH into SalI-digested pBOMBLCRia (*amiA*), lower case for plasmid overlap construction |
| *amiA*_fw | GACGAGGGAACTGCAGAAAG | qPCR primer |
| *amiA*_rev | CATCCGTAGCCCTTGTCATTAT | qPCR primer |
| *euo*_fw | CGAAGACTACTCGTTGGGAAATA | qPCR primer |
| *euo*_rev | AACAGAAGCTCTCCTTGATAAGT | qPCR primer |
| CT_268_XmaI_52b_for | GCGCGCCCCGGGAGGGGTATCAGATCTTCAAAC | Cloning of *ct268* into pET-52b |
| CT_268-SP_XmaI_52B_for | GCGCGCCCCGGGGCGGGGATTCCTAAGG | Cloning of *ct268* into pET-52b (residues 148-780) |
| CT_268_EagI_52b_rev | GCGCGCCGGCCGTTATTTATGCACTTTTTTTGCTC | Cloning of *ct268* into pET-52b |
| *ct268*H67A_for | TGAACTTATAGTCATAGATCCTGGTGCTGGCGGTAAAGACG | *ct268* mutagenesis |
| *ct268*H67A_rev | CGTCTTTACCGCCAGCACCAGGATCTATGACTATAAGTTCA | *ct268* mutagenesis |
| *ct268*H136A_for | GATGTATTAGACGAGTGATTACAGGCGATACTAACAAAAACATCAGCCTT | *ct268* mutagenesis |
| *ct268*H136A_rev | AAGGCTGATGTTTTTGTTAGTATCGCCTGTAATCACTCGTCTAATACATC | *ct268* mutagenesis |
| *ct268*E205A_for | CCTGCGGTCCTTGTGGCAACAGGGTTTCTTTCC | *ct268* mutagenesis |
| *ct268*E205A_rev | GGAAAGAAACCCTGTTGCCACAAGGACCGCAGG | *ct268* mutagenesis |
| *ct268*+_pASK3_vec_fw | TGGAGCCACCCGCAGTTC | Linearization of pASK3C |
| *ct268*+_pASK3_vec_rev | TTCTCTATCACTGATAGGGAGTGG | Linearization of pASK3C |
| *ct268*+_pASK3_ins_fw | ATCAGTGATAGAGAAATGAGGGGTATCAGATCTTCAAACA | Amplification of *ct268* |
| *ct268*_pASK3_ins_rev | CTGCGGGTGGCTCCATTTATGCACTTTTTTTGCTCCTGA | Amplification of *ct268* |
| *ct268*ΔSP_pASK3_ins_fw | ATCAGTGATAGAGAAGCGGGGATTCCTAAGGTAAGT | Amplification of *ct268* (residues 148-780) |
| *murE*_fw | CCTTCAAGGCATCTGGATGTAA | qPCR primer |
| *murE*_rev | CTTCTTCCTGAACGCTCCATAA | qPCR primer |
| *omcB*_fw | CGGTAGGATCTCCCTATCCTATT | qPCR primer |
| *omcB*_rev | CGAACTCTGCTTCACATGGTA | qPCR primer |
| pASK3_*amiA*Ct_ins_fw | ATCAGTGATAGAGAAATGAGGGGTATCAGATCTTCAAACA | Amplification of *ct268* |
| pASK3_*amiA*Ct_ins_rev | CTGCGGGTGGCTCCATTTATGCACTTTTTTTGCTCCTGA | Amplification of *ct268* |
| pASK3_*amiA*Ct_vec_fw | TGGAGCCACCCGCAGTTC | Linearization of pASK3 |
| pASK3_*amiA*Ct_vec_rev | TTCTCTATCACTGATAGGGAGTGG | Linearization of pASK3 |

**Supplemental Table 4: gBlock sequences**

| **gBlock name** | **Sequence (5‘-3‘)** | **Features** | **Usage** |
| --- | --- | --- | --- |
| *amiA*-targeting gRNA gBlock cassette | tgtgaaagtgggtcttaagacgtcggtactgcatgtgacgcacgtagatcatgca*TTCACCGGTGGAGACGGTTTTCTTATAATGACACC***AAGTTCATTCCTACTTACCTT**GTTTTAGTACTCTGGAAACAGAATCTACTAAAACAAGGCAAAATGCCGTGTTTATCTCGTCAACTTGTTGGCGAGATTTTTCAAATAAAACGAAAGGCTCAGTCGAAAGACTGGGCCTTTCGTTTTATcaacagcggtctactgaatctgagctagtgcgtgatataattaaaattatattca | Lower case for plasmid overlap and spacer, *italicized* for P_dnaKmut_ promoter sequence, **bold** for amiA-targeting sequence, underlined for gRNA scaffold, Upper case for rrnB1 terminator | Insert into BamHI-digested pBOMBL-Sa_dCas9vaa::L2 plasmid |
| Non-targeting gRNA gBlock cassette | tgtgaaagtgggtcttaagacgtcggtactgcatgtgacgcacgtagatcatgca*TTCACCGGTGGAGACGGTTTTCTTATAATGACACC***ACCGAGTTGCCCGTTAAAGTA**GTTTTAGTACTCTGGAAACAGAATCTACTAAAACAAGGCAAAATGCCGTGTTTATCTCGTCAACTTGTTGGCGAGATTTTTCAAATAAAACGAAAGGCTCAGTCGAAAGACTGGGCCTTTCGTTTTATcaacagcggtctactgaatctgagctagtgcgtgatataattaaaattatattca | Lower case for plasmid overlap and spacer, *italicized* for P_dnaKmut_ promoter sequence, **bold** for non-targeting sequence, underlined for gRNA scaffold, Upper case for rrnB1 terminator | Insert into BamHI-digested pBOMBL-Sa_dCas9vaa::L2 plasmid |

REFERENCES

31. Klöckner A, Otten C, Derouaux A, Vollmer W, Bühl H, De Benedetti S, Münch D, Josten M, Mölleken K, Sahl H-G, Henrichfreise B. 2014. AmiA is a penicillin target enzyme with dual activity in the intracellular pathogen *Chlamydia pneumoniae*. Nat Commun 5:4201. <https://doi.org/10.1038/ncomms5201>

60. Miroux B, Walker JE. 1996. Over-production of proteins in *Escherichia coli*: mutant hosts that allow synthesis of some membrane proteins and globular proteins at high levels. J Mol Biol 260:289–298. <https://doi.org/10.1006/jmbi.1996.0399>
